# Synthesis, crystal structure and photophysical properties of a dinuclear Mn^II^ complex with 6-(di­ethyl­amino)-4-phenyl-2-(pyridin-2-yl)quinoline

**DOI:** 10.1107/S2056989024006042

**Published:** 2024-06-28

**Authors:** Hai Le Thi Hong, Duong Hoang Tuan, Anh Nguyen Duc, Hien Nguyen, Luc Van Meervelt

**Affiliations:** a Department of Chemistry, Hanoi National University of Education, 136 Xuan Thuy, Cau Giay, Hanoi, Vietnam; b Department of Chemistry, KU Leuven, Biomolecular Architecture, Celestijnenlaan 200F, Leuven (Heverlee), B-3001, Belgium; Vienna University of Technology, Austria

**Keywords:** crystal structure, Mn^II^ complex, dinuclear complex, aggregation-induced emission

## Abstract

A new quinoline derivative, namely, 6-(di­ethyl­amino)-4-phenyl-2-(pyridin-2-yl)quinoline, C_24_H_23_N_3_ (**QP**), and its Mn^II^ complex aqua-1κ*O*-di-μ-chlorido-1:2κ^4^*Cl*:*Cl*-di­chlorido-1κ*Cl*,2κ*Cl*-bis­[6-(di­ethyl­amino)-4-phenyl-2-(pyridin-2-yl)quinoline]-1κ^2^*N*^1^,*N*^2^;2κ^2^*N*^1^,*N*^2^-dimanganese(II), [Mn_2_Cl_4_(C_24_H_23_N_3_)_2_(H_2_O)] (**MnQP**), were synthesized. Their compositions have been determined with ESI-MS, IR, and ^1^H NMR spectroscopy. The crystal-structure determination of **MnQP** revealed a dinuclear complex with a central four-membered Mn_2_Cl_2_ ring. Both Mn^II^ atoms bind to an additional Cl atom and to two N atoms of the **QP** ligand. One Mn^II^ atom expands its coordination sphere with an extra water mol­ecule, resulting in a distorted octa­hedral shape. The second Mn^II^ atom shows a distorted trigonal–bipyramidal shape. The UV–vis absorption and emission spectra of the examined compounds were studied. Furthermore, when investigating the aggregation-induced emission (AIE) properties, it was found that the fluorescent color changes from blue to green and eventually becomes yellow as the fraction of water in the THF/water mixture increases from 0% to 99%. In particular, these color and intensity changes are most pronounced at a water fraction of 60%. The crystal structure contains disordered solvent mol­ecules, which could not be modeled. The SQUEEZE procedure [Spek (2015 ▸). *Acta Cryst.* C**71**, 9–18] was used to obtain information on the type and qu­antity of solvent mol­ecules, which resulted in 44 electrons in a void volume of 274 Å^3^, corresponding to approximately 1.7 mol­ecules of ethanol in the unit cell. These ethanol mol­ecules are not considered in the given chemical formula and other crystal data.

## Abstract 2

The two Mn^II^ atoms in the complex display a different coordination number, *viz*. five with a distorted trigonal–bipyramidal Cl_3_N_2_ and six with a distorted octa­hedral Cl_3_N_2_O coordination set.

## 1. Chemical context

Among heterocyclic compounds, quinoline derivatives are of great inter­est because they have many inter­esting properties in terms of both biological and photophysical properties. For example, compounds consisting of quinine, chloro­quine, amidiaquine and primaquine have anti­malarial activity; 8-hy­droxy­quinoline is used to produce pesticides; some derivatives of quinoline are capable of emitting visible light (Sales *et al.*, 2015 ▸; dos Santos *et al.*, 2017 ▸). Currently, quinoline derivatives synthesized from multicomponent reactions including an aniline derivative, an aldehyde and a phenyl­alkyne with green catalysts are a trend that is receiving more attention due to a one-pot reaction with high yields. Moreover, by changing substituents in the components, it is possible to create many new derivatives of quinoline containing both aryl rings and long π-conjugation systems, and their application can be expanded (Sales *et al.*, 2015 ▸; Sharghi *et al.*, 2016 ▸). There are also many quinoline derivatives that have some inter­esting photophysical properties such as metal-ion recognition (Wang *et al.*, 2020 ▸; Hojitsiriyanont *et al.*, 2021 ▸; Mohanasundaram *et al.*, 2021 ▸) or aggregation-induced emission (AIE) properties (Zhang *et al.*, 2019 ▸; Shen *et al.*, 2021 ▸; Hussain *et al.*, 2022 ▸). In addition, some quinoline derivatives have been designed that contain electron-donating atoms, N,N-donor ligands, capable of forming chelate complexes with transition-metal ions. Complexes of this type of ligands not only have more diverse structures, but also a large number of superior properties compared to the free ligands, such as higher anti­cancer activities (Shakir *et al.*, 2015 ▸; Wang *et al.*, 2017 ▸; Hu *et al.*, 2018 ▸) or better optical properties (Pathaw *et al.*, 2021 ▸).
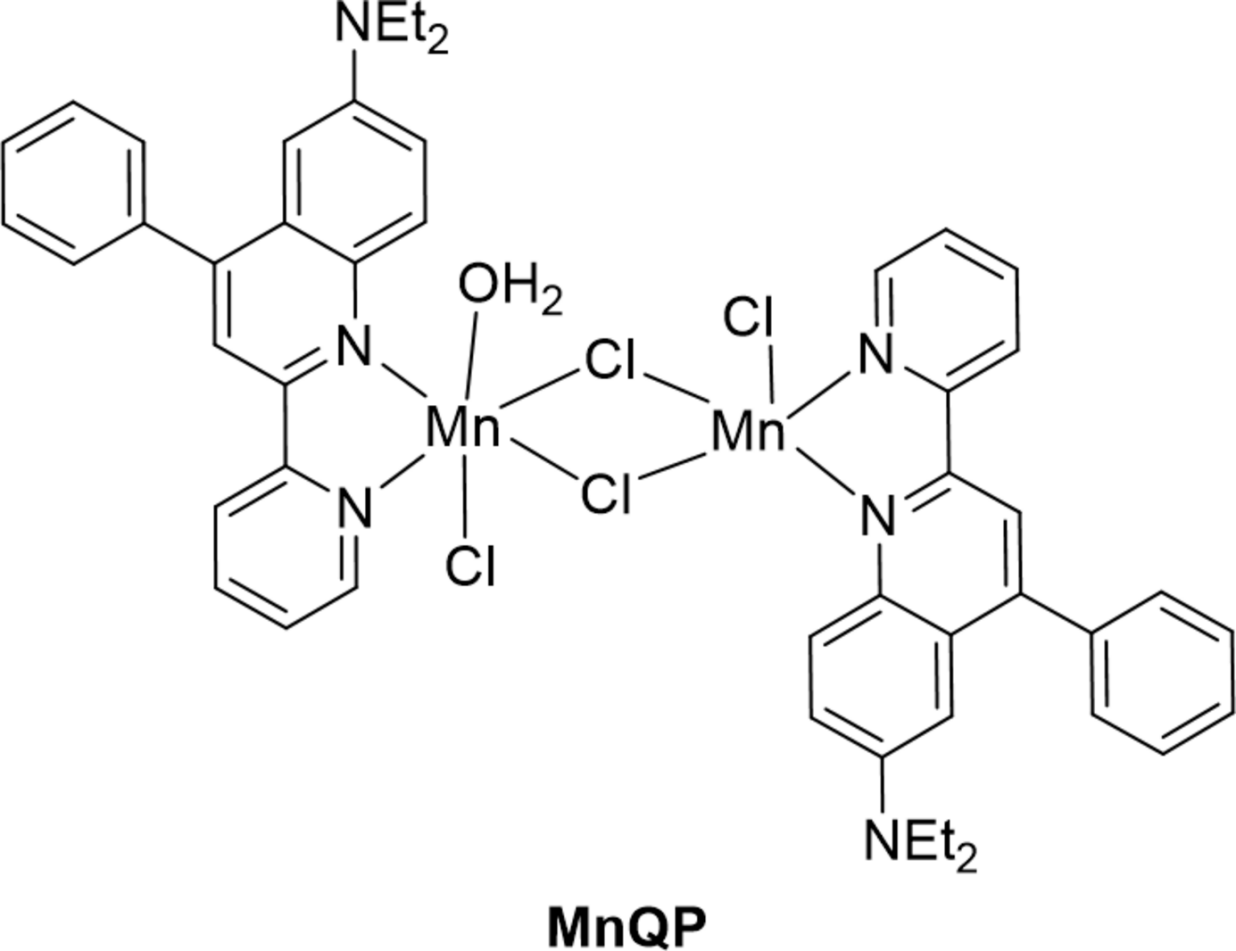


**Figure 1:**
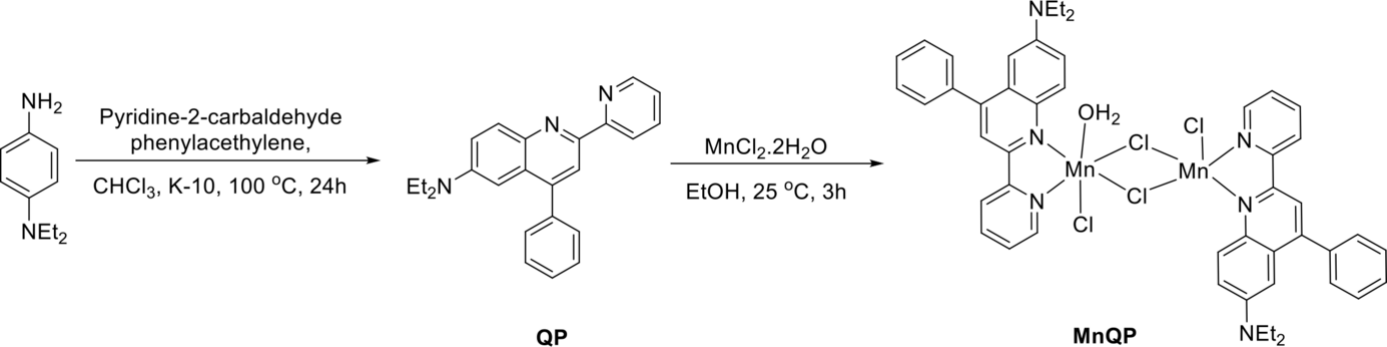
Synthesis scheme of **QP** and title compound **MnQP**.

In this report, a new quinoline derivative, 6-(*N*,*N*-di­ethyl­amine)-4-phenyl-2(pyridin-2-yl)quinoline (**QP**), was synthesized *via* a one-pot reaction involving 4-*N*,*N*-di­ethylamine­aniline, pyridine-2-carbaldehyde and phenyl­acetyl­ene. The green catalyst used in this synthesis was montmorillonite (K-10; Fig. 1 ▸). For this compound, two electron-withdrawing groups – pyridine and phenyl – were introduced at positions C2 and C4 of the quinoline ring. In addition, an electron-donating group, *N,N*-di­ethyl­amino (–NEt_2_), was also incorporated to create an electron push–pull effect. This effect contributes to an intra­molecular charge transfer (ICT) during excitation *via* photon absorption. Furthermore, the organic compound contains two N-donor atoms from the quinoline and pyridine rings. As a result, the ligand can form five-membered ring chelate complexes with transition-metal ions. More specifically, Mn^II^, with a *d*^5^ semi-saturated electronic configuration, is able to form complexes with various coord­ination numbers, ranging from 4 to 7 (Jin *et al.*, 2011 ▸; Li *et al.*, 2011 ▸; Konar *et al.*, 2011 ▸; Wang *et al.*, 2017 ▸; Sääsk *et al.*, 2024 ▸). Therefore, when Mn^II^ inter­acts with the **QP** ligand, mononuclear and polynuclear complexes with different coordination numbers can be expected. The structure of the product complex, referred to as **MnQP**, was determined using single-crystal X-ray diffraction. Furthermore, the photophysical and aggregation-induced emission (AIE) properties of both **QP** and **MnQP** were investigated using UV–vis absorption and emission spectra.

## 2. Structural commentary

**Figure 2:**
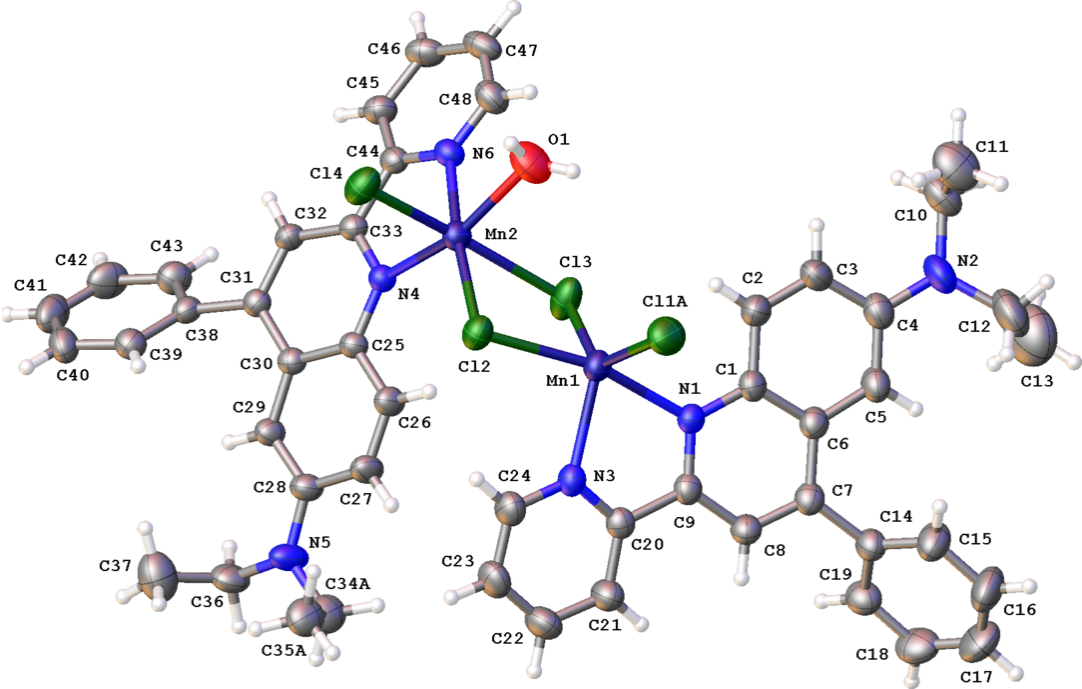
The mol­ecular structure of **MnQP** with complete labeling of non-hydrogen atoms. Displacement ellipsoids are shown at the 30% probability level. For the Cl atom Cl1 and ethyl group C34–C35, only the part with the higher occupancy is shown.

**MnQP** crystallizes in the triclinic space group *P*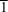 with one complex mol­ecule in the asymmetric unit (Fig. 2 ▸). The complex contains two Mn^II^ atoms, two **QP** ligands (denoted *A* and *B*, containing atoms N1 and N4, respectively), four chlorine atoms and one water mol­ecule. Chlorine atom Cl1 is disordered over two positions with a refined occupancy ratio of Cl1*A*:Cl1*B* = 0.680 (8):0.320 (8). For the disordered ethyl group C34–C35, the occupancy ratio refined to 0.878 (4):0.122 (4). The crystal structure contains disordered solvent mol­ecules, which could not be modeled. The SQUEEZE procedure (Spek, 2015 ▸) was used to obtain information on the type and qu­antity of solvent mol­ecules, which resulted in 44 electrons in a void volume of 274 Å^3^, corresponding to approximately 1.7 mol­ecules of ethanol in the unit cell.

Two bridging chlorine atoms (Cl2, Cl3) connect the two central Mn^II^ atoms to form a four-membered rhomb-shaped ring. The metal⋯metal distance is 3.7412 (6) Å. Both Mn^II^ atoms have a different coordination environment, fivefold for Mn1 and sixfold for Mn2. The coordination sphere of Mn1 is best described as distorted trigonal–bipyramidal. The equatorial positions are occupied by nitro­gen atom N3 at a distance of 2.215 (2) Å, and two chlorine atoms Cl1 and Cl3 at distances of, respectively, 2.382 (2) (for Cl1*A*), 2.337 (4) (for Cl1*B*) and 2.4501 (8) Å. The axial positions are occupied by chlorine atom Cl2 at a distance of 2.4974 (7) Å and nitro­gen atom N1 at a distance of 2.286 (2) Å. The Mn2 ion exhibits a distorted octa­hedral coordination sphere, with the equatorial plane formed by three chlorine atoms Cl2, Cl3 and Cl4 at distances of 2.6269 (8), 2.5838 (8) and 2.4354 (8) Å, respectively, and one nitro­gen atom N6, at a distance of 2.257 (2) Å. One axial position is occupied by water oxygen atom O1 at a distance of 2.213 (2) Å, the other by nitro­gen atom N4 at a distance of 2.3087 (19) Å.

The planar quinoline ring in ligand *A* (r.m.s. deviation = 0.014 Å) makes a dihedral angle of 9.46 (8)° with pyridine ring N3/C20–C24 and 54.84 (10)° with phenyl ring C14–C19. In ligand *B*, the quinoline ring (r.m.s. deviation = 0.061 Å) makes a significantly larger dihedral angle with the pyridine ring N6/C44–C48 [23.39 (7)°] and a smaller one with phenyl ring C38–C43 [50.15 (8)°]. The two quinoline rings are mutually inclined at an angle of 53.07 (6)°. The sum of the bond angles around N2 [358.0 (5)°] and N5 [360.0 (3)°] indicate *sp*^2^ hybridization.

## 3. Supra­molecular features

**Figure 3:**
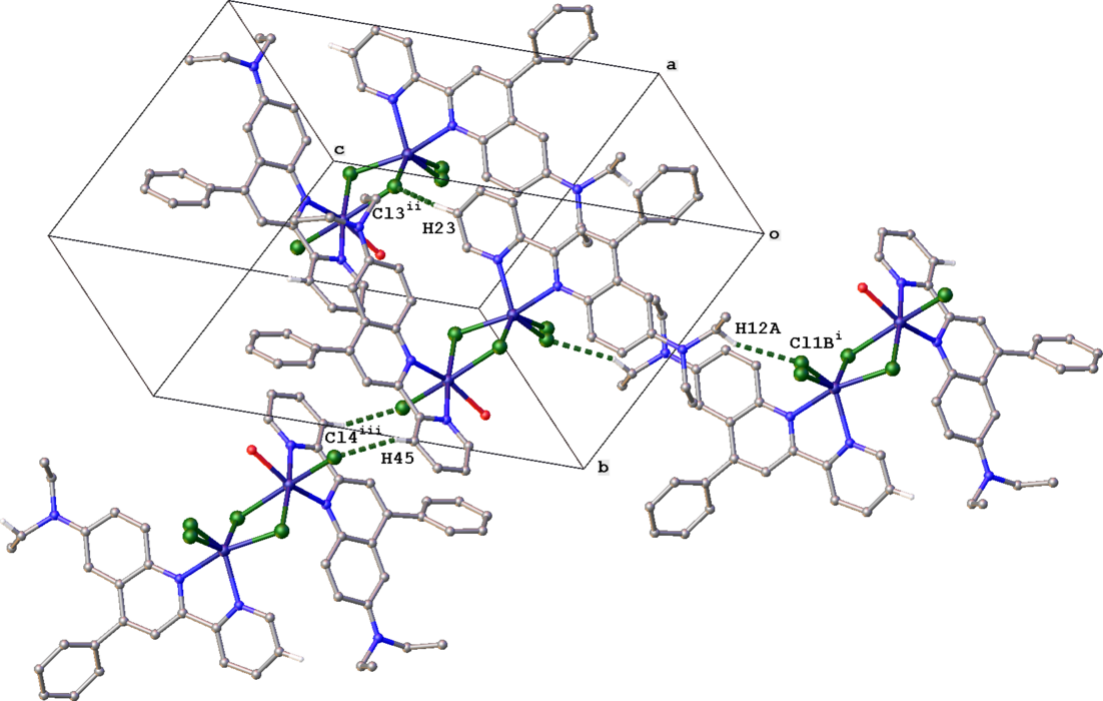
Packing diagram for **MnQP** showing C—H⋯Cl inter­actions (green lines) between mol­ecules. For clarity, only those H atoms involved in hydrogen bonding are shown. Symmetry codes are given in Table 1 ▸.

**Figure 4:**
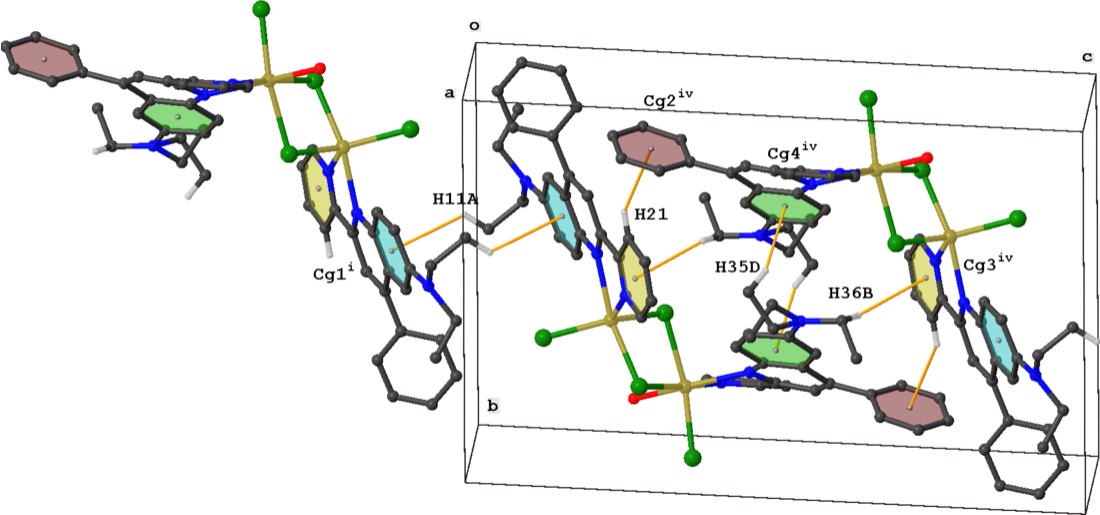
Packing diagram for **MnQP** showing the C—H⋯π inter­actions (orange lines) between mol­ecules. For clarity, only those H atoms involved in the inter­actions are shown. Symmetry codes are given in Table 1 ▸.

The crystal packing of **MnQP** is characterized by C—H⋯Cl and C—H⋯π inter­actions. Inversion dimers are formed by C12—H12*A*⋯Cl1*B* and C45—H45⋯Cl4 inter­actions. Both dimers are part of slabs forming chains parallel to the *a* axis through C23—H23⋯Cl3 inter­actions (Fig. 3 ▸, Table 1 ▸). The packing is further stabilized by four different types of C—H⋯π inter­actions (Fig. 4 ▸, Table 1 ▸).

The hydrogen atoms of water mol­ecule O1 are not involved in hydrogen-bonding inter­actions. Significant π–π stacking inter­actions between rings of neighboring mol­ecules were not observed in this structure.

## 4. Database survey

A search of the Cambridge Structural Database (CSD, Version 5.45, update of March 2024; Groom *et al.*, 2016 ▸) indicated 347 compounds incorporating a four-membered Mn_2_Cl_2_ moiety. Of these compounds, 115 also have two N atoms that bond to the each Mn^II^ atom. The number of similar compounds further reduces to 69 when each Mn^II^ atom bonds to an additional Cl atom. Adding an additional O atom to one of the Mn^II^ atoms results in 12 complexes, all of which exhibit a coordination number of six with a distorted octa­hedral coordination environment for both Mn^II^ atoms, and a (pseudo) inversion center at the center of the Mn_2_Cl_2_ ring. For four complexes, the O atom is part of a water mol­ecule, where the Mn—O distance varies between 2.141 and 2.274 Å [2.323 (2) Å in **MnQP**].

## 5. Photophysical properties

**Figure 5:**
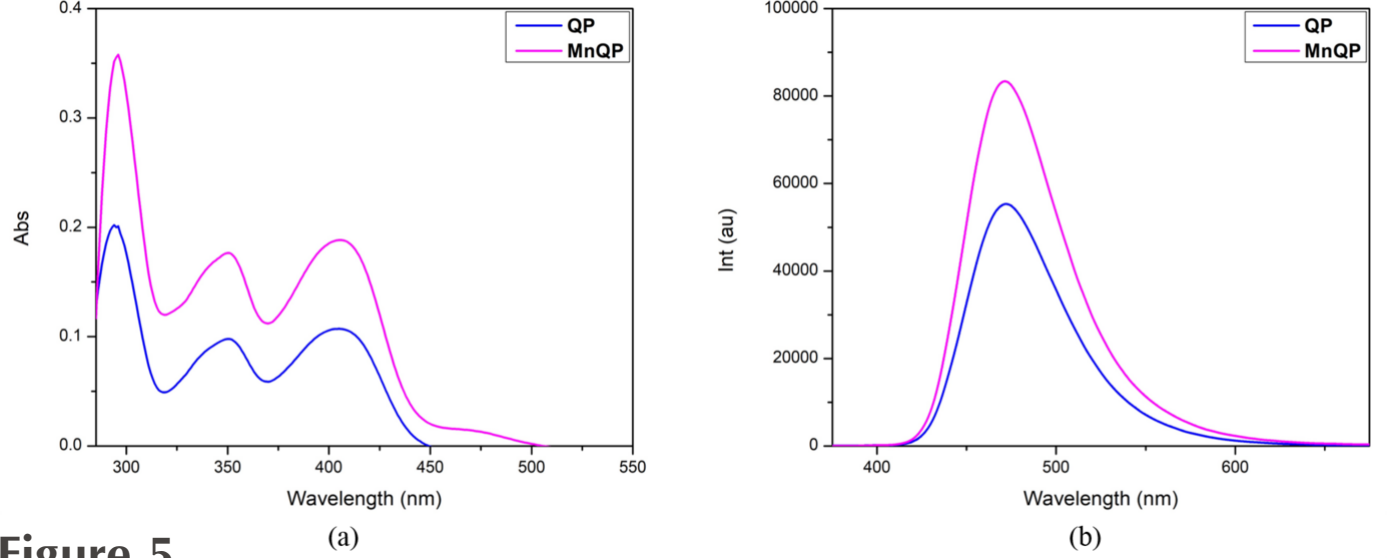
(*a*) UV–vis absorption and (*b*) emission spectra of **QP** and **MnQP** (10 µ*M* in THF, *λ*_ex_ = 360 nm).

The UV–vis absorption and emission spectra of **QP** and **MnQP** (10 µ*M* in THF) are shown in Fig. 5 ▸ and numerical data in Table 2 ▸. In the UV–vis spectra (Fig. 5 ▸*a*), both **QP** and **MnQP** exhibit three absorption bands with maxima at 294 nm, 351 nm, and 405 nm. These bands are attributed to the *n*→π^*^ and π→π^*^ transitions of the fused aromatic heterocycle. In the emission spectra (Fig. 5 ▸*b*), both the ligand and the complex emit light with a band at 472 nm, corresponding to blue light. Although the maximum absorption and emission wavelength do not change significantly between the ligand and the complex, the emission intensity of the complex is higher than that of the free ligand. This enhancement can be explained by the *d*^5^ electronic configuration of the central Mn^II^ ion, which forbids absorption of radiation in the visible range according to the Laporte rules. Additionally, the coordination of Mn^II^ with the ligand through two heterocyclic N atoms reduces rotation of the pyridine ring, leading to an increase in emission intensity from 55338 a.u. to 83395 a.u. compared to the free ligand.

**Figure 6:**
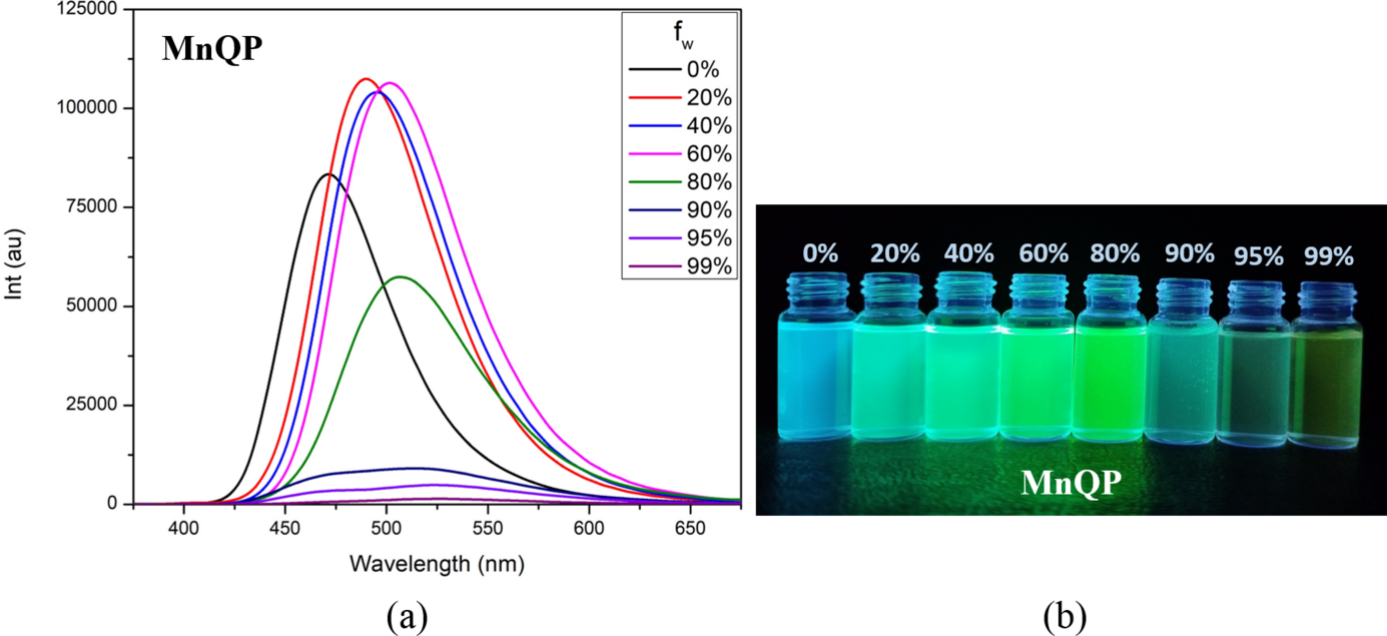
(*a*) Emission spectra and (*b*) fluorescent color change of **MnQP** with a concentration of 10 µ*M* in different fractions of water in a THF–water mixture.

The aggregation-induced emission (AIE) properties of **QP** and **MnQP** were investigated by recording photoluminescence (PL) spectra in THF/water mixtures with different water fractions (fw) at a concentration of 10 µ*M*. The results show that their fluorescent color changes from blue to green and finally turns yellow under 365 nm UV light when the water fraction increases from 0% to 99%. For the **QP** ligand, the color and intensity changes are most pronounced at a 60% water ratio (see Fig. S6 in the electronic supporting information, ESI), and the same trend is observed for the **MnQP** complex (Fig. 6 ▸). This behavior can be explained by the following factors. As the water fraction in the THF–water mixture increases, the solubility of both the ligand and the complex decreases. This reduction in solubility leads to shorter distances between mol­ecules, which in turn promotes π–π inter­actions between adjacent mol­ecules. This inter­action changes the electron density within the mol­ecules, resulting in changes in the emission peak and intensity (Hong *et al.*, 2009 ▸).

## 6. Synthesis and crystallization

**Synthesis of 6-(*****N***,***N*****-di­ethyl­amine)-4-phenyl-2(pyridin-2-yl)quinoline (QP)**

To a mixture of 4-*N,N*-di­ethyl­amine­aniline (196.8 mg, 1.2 mmol), pyridine-2-carbaldehyde (128.4 mg, 1.2 mmol), and phenyl­acetyl­ene (102.0 mg, 1.0 mmol) were added montmorillonite (K-10) (500 mg) and chloro­form (1 ml). The resulting reaction mixture was stirred continuously at 373 K. After 24 h, the reaction mixture was cooled down to room temperature, and extracted three times with ethyl­acetate/water (*v*/*v* = 1:1). The collected organic phase was dried over anhydrous sodium sulfate and concentrated under reduced pressure using a rotatory evaporator to remove the solvent. The residue was then adsorbed on silicagel and purified by silica gel column chromatography with ethyl­ acetate/*n*-hexane (*v*/*v* = 1:10) to obtain **QP** as a dark-orange solid. The isolated yield of this cyclization reaction is 65%. The product is moderately soluble in ethanol, THF, CHCl_3_, and DMSO. ESI–MS: 356.3 (100%) = [QP + H]^+. 1^H NMR (600 MHz, CDCl_3_, δ ppm): 1.16 (6H, ^3^*J* = 7.2 Hz, *t*, 2 CH_3_), 3.80 (4H, ^3^*J* = 7.2 Hz, *q*, 2 CH_2_), 6.90 (1H, ^4^*J* = 3.0 Hz, d, Ar-H), 7.27 (1H, ^3^*J* = 6.0 Hz, ^4^*J* = 1.2 Hz, *td*, Ar-H), 7.32 (1H, ^3^*J* = 9.6 Hz, ^4^*J* = 3.0 Hz, *dd*, Ar-H), 7.45 (1H, ^3^*J* = 7.2 Hz, *d*, Ar-H), 7.49 (2H, ^3^*J* = 6.6 Hz, *t*, Ar-H), 7.60 (2H, ^3^*J* = 7.2 Hz, *d*, Ar-H), 7.82 (1H, ^3^*J* = 7.8 Hz, ^4^*J* = 1.8 Hz, *td*, Ar-H), 8.07 (1H, ^3^*J* = 9.6 Hz, *d*, Ar-H), 8.35 (1H, *s*, Ar-H), 8.59 (1H, ^3^*J* = 7.8 Hz, *d*, Ar-H), 8.67 (1H, ^3^*J* = 6.0 Hz, *d*, Ar-H). IR (KBr, cm^−1^): 2965 (ν_C—H ar­yl_), 1615, 1585 (ν_C=C ar­yl_), 1504, 1435 (ν_C=N ar­yl_).

ESI–MS, FT–IR and ^1^H NMR spectra of **QP** are given in Figs. S1, S2 and S3, respectively, in the ESI.

**Synthesis of [Mn_2_(QP)_2_Cl_4_(H_2_O)] (MnQP)**

MnCl_2_·2H_2_O (35.64 mg, 0.22 mmol) was added to a **QP** solution (70.6 mg, 0.2 mmol in 3 ml of ethanol). The resulting mixture was stirred continuously at room temperature for 3 h and became dark yellow. The solution was evaporated slowly for 48 h to obtain yellow crystals of **MnQP**. The crystals were then filtered and washed with acetone. The yield was about 52%. The crystals are moderately soluble in ethanol, THF, CHCl_3_ and DMSO. ESI–MS: 729.3 (65%) = [Mn_2_(QP)_2_Cl_4_(H_2_O)-QP+2DMSO-H_2_O-H]^+^; 937.8 (20%) = [Mn_2_(QP)_2_Cl_4_(H_2_O)-Cl]^+^. IR (KBr, cm^−1^): 3407 (ν_O—H_ H_2_O), 2971 (ν_C—H ar­yl_), 1614, 1599 (ν_C=C ar­yl_), 1506, 1483 (ν_C=N ar­yl_).

ESI–MS and FT–IR spectra of **MnQP** are given in Figs. S4 and S5, respectively, in the ESI.

## 7. Refinement

Crystal data, data collection and structure refinement details are summarized in Table 3 ▸. Hydrogen atoms were included as riding contributions in idealized positions with isotropic displacement parameters *U*_iso_(H) = 1.2 *U*_eq_(C) (1.5 for methyl groups). The Cl1 atom and ethyl group C34–C35 were found to be disordered over two positions with refined occupancies of 0.680 (8) and 0.320 (8) for Cl1, and 0.878 (4) and 0.122 (4) for ethyl group C34–C35. The H atoms of the water mol­ecule were located in a difference electron-density map and refined with *U*_iso_(H) = 1.5*U*_eq_(O) and O—H distances restrained to 0.82 Å. RIGU and DELU restraints were used for atoms N2, Cl2 and Cl3 to impose reasonable relative motion of these atoms. Additional electron density was localized in voids (274 Å^3^ total potential accessible volume) summing up to 44 electrons, which corresponds to approximately 1.7 mol­ecules of ethanol per unit cell. The electron density associated with the disordered ethanol mol­ecules was removed with the SQUEEZE (Spek, 2015 ▸) routine in *PLATON* (Spek, 2020 ▸). These ethanol mol­ecules are not considered in the given chemical formula and other crystal data.

## Supplementary Material

Crystal structure: contains datablock(s) I. DOI: 10.1107/S2056989024006042/wm5726sup1.cif

Structure factors: contains datablock(s) I. DOI: 10.1107/S2056989024006042/wm5726Isup2.hkl

ESI-MS, FT-IR and H-NMR spectra. DOI: 10.1107/S2056989024006042/wm5726sup3.pdf

CCDC reference: 2364423

Additional supporting information:  crystallographic information; 3D view; checkCIF report

## Figures and Tables

**Table 1:** Hydrogen-bond geometry (Å, °) *Cg1*–*Cg*4 are the centroids of the C1–C6, C38–C43, N3/C20–C24 and C25–C30 rings, respectively.

*D*—H⋯*A*	*D*—H	H⋯*A*	*D*⋯*A*	*D*—H⋯*A*
C12—H12*A*⋯Cl1*B*^i^	0.97	2.81	3.734 (7)	158
C23—H23⋯Cl3^ii^	0.93	2.74	3.567 (3)	149
C45—H45⋯Cl4^iii^	0.93	2.69	3.563 (3)	157
C11—H11*A*⋯*Cg*1^i^	0.96	2.97	3.585 (4)	123
C21—H21⋯*Cg*2^iv^	0.93	2.89	3.642 (3)	139
C36—H36*B*⋯*Cg*3^iv^	0.97	2.96	3.845 (3)	153
C35*B*—H35*D*⋯*Cg*4^iv^	0.96	2.84	3.45 (2)	122

Symmetry codes: (i) 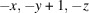; (ii) 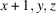; (iii) 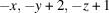; (iv) 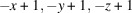.

**Table 2:** Photophysical data for QP and MnQP (in THF, 10 µ*M*)

Compound	Absorption	Emission		Stokes shift
	λ_*ABS*_(nm) / ɛ (10^−3^*M*^−1^.cm^−1^)	λ_em_ (nm)	Intensity (a.u.)	Δν (cm^−1^)
**QP**	294 (21); 351 (10); 405 (11)	472	55338	7303
**MnQP**	294 (36); 351 (18); 405 (19)	472	83395	7340

**Table 3:** Experimental details

Crystal data	
Chemical formula	[Mn_2_Cl_4_(C_24_H_23_N_3_)_2_(H_2_O)]
*M*_r_	976.60
Crystal system, space group	Triclinic, *P* 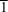
Temperature (K)	294
*a*, *b*, *c* (Å)	8.7491 (2), 13.2133 (3), 21.3793 (5)
α, β, γ (°)	88.914 (2), 82.290 (2), 88.989 (2)
*V* (Å^3^)	2448.48 (10)
*Z*	2
Radiation type	Mo *K*α
μ (mm^−1^)	0.78
Crystal size (mm)	0.4 × 0.15 × 0.05

Data collection	
Diffractometer	SuperNova, Single source at offset/far, Eos
Absorption correction	Multi-scan (*CrysAlis PRO*; Rigaku OD, 2024 ▸)
*T*_min_, *T*_max_	0.606, 1.000
No. of measured, independent and observed [*I* > 2σ(*I*)] reflections	50282, 9973, 7946
*R*_int_	0.034
(sin θ/λ)_max_ (Å^−1^)	0.625

Refinement	
*R*[*F*^2^ > 2σ(*F*^2^)], *wR*(*F*^2^), *S*	0.044, 0.139, 0.86
No. of reflections	9973
No. of parameters	560
No. of restraints	18
H-atom treatment	H atoms treated by a mixture of independent and constrained refinement
Δρ_max_, Δρ_min_ (e Å^−3^)	0.86, −0.47

Computer programs: *CrysAlis PRO* (Rigaku OD, 2024 ▸), *SHELXT* (Sheldrick, 2015*a* ▸), *SHELXL* (Sheldrick, 2015*b* ▸) and *OLEX2* (Dolomanov *et al.*, 2009 ▸).

## supplementary crystallographic information

### Aqua-1κO-di-µ-chlorido-1:2κ4Cl:Cl-dichlorido-1κCl,2κCl-bis[6-(diethylamino)-4-phenyl-2-(pyridin-2-yl)quinoline]-1κ2N1,N2;2κ2N1,N2-dimanganese(II) Crystal data

**Table:**

[Mn_2_Cl_4_(C_24_H_23_N_3_)_2_(H_2_O)]	*Z* = 2
*M**_r_* = 976.60	*F*(000) = 1008
Triclinic, *P*1	*D*_x_ = 1.325 Mg m^−^^3^
*a* = 8.7491 (2) Å	Mo *K*α radiation, λ = 0.71073 Å
*b* = 13.2133 (3) Å	Cell parameters from 20239 reflections
*c* = 21.3793 (5) Å	θ = 3.6–27.7°
α = 88.914 (2)°	µ = 0.78 mm^−^^1^
β = 82.290 (2)°	*T* = 294 K
γ = 88.989 (2)°	Plate, yellow
*V* = 2448.48 (10) Å^3^	0.4 × 0.15 × 0.05 mm

### Aqua-1κO-di-µ-chlorido-1:2κ4Cl:Cl-dichlorido-1κCl,2κCl-bis[6-(diethylamino)-4-phenyl-2-(pyridin-2-yl)quinoline]-1κ2N1,N2;2κ2N1,N2-dimanganese(II) Data collection

**Table:**

SuperNova, Single source at offset/far, Eos diffractometer	9973 independent reflections
Radiation source: micro-focus sealed X-ray tube, SuperNova (Mo) X-ray Source	7946 reflections with *I* > 2σ(*I*)
Mirror monochromator	*R*_int_ = 0.034
Detector resolution: 15.9566 pixels mm^-1^	θ_max_ = 26.4°, θ_min_ = 3.3°
ω scans	*h* = −10→10
Absorption correction: multi-scan (CrysAlisPro; Rigaku OD, 2024)	*k* = −16→16
*T*_min_ = 0.606, *T*_max_ = 1.000	*l* = −26→26
50282 measured reflections	

### Aqua-1κO-di-µ-chlorido-1:2κ4Cl:Cl-dichlorido-1κCl,2κCl-bis[6-(diethylamino)-4-phenyl-2-(pyridin-2-yl)quinoline]-1κ2N1,N2;2κ2N1,N2-dimanganese(II) Refinement

**Table:**

Refinement on *F*^2^	Primary atom site location: dual
Least-squares matrix: full	Hydrogen site location: mixed
*R*[*F*^2^ > 2σ(*F*^2^)] = 0.044	H atoms treated by a mixture of independent and constrained refinement
*wR*(*F*^2^) = 0.139	*w* = 1/[σ^2^(*F*_o_^2^) + (0.0889*P*)^2^ + 2.333*P*] where *P* = (*F*_o_^2^ + 2*F*_c_^2^)/3
*S* = 0.86	(Δ/σ)_max_ = 0.001
9973 reflections	Δρ_max_ = 0.86 e Å^−^^3^
560 parameters	Δρ_min_ = −0.47 e Å^−^^3^
18 restraints	

### Aqua-1κO-di-µ-chlorido-1:2κ4Cl:Cl-dichlorido-1κCl,2κCl-bis[6-(diethylamino)-4-phenyl-2-(pyridin-2-yl)quinoline]-1κ2N1,N2;2κ2N1,N2-dimanganese(II) Special details

**Table:**

Geometry. All esds (except the esd in the dihedral angle between two l.s. planes) are estimated using the full covariance matrix. The cell esds are taken into account individually in the estimation of esds in distances, angles and torsion angles; correlations between esds in cell parameters are only used when they are defined by crystal symmetry. An approximate (isotropic) treatment of cell esds is used for estimating esds involving l.s. planes.

### Aqua-1κO-di-µ-chlorido-1:2κ4Cl:Cl-dichlorido-1κCl,2κCl-bis[6-(diethylamino)-4-phenyl-2-(pyridin-2-yl)quinoline]-1κ2N1,N2;2κ2N1,N2-dimanganese(II) Fractional atomic coordinates and isotropic or equivalent isotropic displacement parameters (Å^2^)

**Table:**

	*x*	*y*	*z*	*U*_iso_*/*U*_eq_	Occ. (<1)
Mn1	0.35314 (5)	0.65406 (3)	0.22302 (2)	0.03955 (12)	
Cl1A	0.3891 (5)	0.7046 (2)	0.11458 (10)	0.0598 (4)	0.680 (8)
Cl1B	0.3379 (8)	0.7186 (4)	0.1215 (2)	0.0598 (4)	0.320 (8)
O1	0.0739 (3)	0.8996 (2)	0.25271 (12)	0.0689 (7)	
H1A	0.101 (5)	0.9574 (15)	0.243 (2)	0.103*	
H1B	0.072 (6)	0.866 (3)	0.2203 (14)	0.103*	
N1	0.3023 (2)	0.49080 (16)	0.20012 (10)	0.0365 (5)	
C1	0.1824 (3)	0.4612 (2)	0.17034 (12)	0.0383 (5)	
Mn2	0.17320 (4)	0.84639 (3)	0.33759 (2)	0.03688 (12)	
Cl2	0.44135 (8)	0.81104 (5)	0.26973 (3)	0.04346 (16)	
N2	−0.1917 (3)	0.3857 (2)	0.07467 (15)	0.0666 (8)	
C2	0.0623 (3)	0.5316 (2)	0.16218 (14)	0.0482 (7)	
H2	0.066162	0.596571	0.177875	0.058*	
Cl3	0.12894 (9)	0.66307 (6)	0.30488 (4)	0.0539 (2)	
N3	0.5448 (2)	0.55925 (16)	0.25097 (10)	0.0388 (5)	
C3	−0.0584 (3)	0.5064 (2)	0.13192 (14)	0.0506 (7)	
H3	−0.135308	0.554530	0.127541	0.061*	
Cl4	0.25143 (9)	1.01982 (6)	0.35077 (4)	0.05338 (19)	
N4	0.1805 (2)	0.79905 (15)	0.44166 (9)	0.0314 (4)	
C4	−0.0703 (3)	0.4089 (2)	0.10692 (13)	0.0475 (7)	
N5	0.6580 (3)	0.5855 (2)	0.53537 (11)	0.0541 (6)	
C5	0.0438 (3)	0.3380 (2)	0.11605 (13)	0.0461 (6)	
H5	0.035832	0.272545	0.101729	0.055*	
N6	−0.0657 (2)	0.86758 (16)	0.39068 (10)	0.0374 (5)	
C6	0.1720 (3)	0.36243 (19)	0.14655 (12)	0.0379 (5)	
C7	0.2938 (3)	0.29213 (19)	0.15545 (12)	0.0391 (6)	
C8	0.4082 (3)	0.32372 (19)	0.18789 (12)	0.0392 (6)	
H8	0.485393	0.278036	0.196120	0.047*	
C9	0.4120 (3)	0.42339 (19)	0.20908 (11)	0.0360 (5)	
C10	−0.3167 (4)	0.4582 (3)	0.06752 (15)	0.0582 (8)	
H10A	−0.338472	0.496987	0.105847	0.070*	
H10B	−0.408843	0.421213	0.062418	0.070*	
C11	−0.2811 (5)	0.5301 (3)	0.01241 (18)	0.0830 (12)	
H11A	−0.260119	0.492405	−0.025818	0.125*	
H11B	−0.192547	0.569133	0.017930	0.125*	
H11C	−0.368005	0.574673	0.009902	0.125*	
C12	−0.1760 (5)	0.2956 (3)	0.0310 (2)	0.0854 (12)	
H12A	−0.225658	0.311567	−0.006007	0.103*	
H12B	−0.067625	0.281812	0.017003	0.103*	
C13	−0.2449 (7)	0.2072 (4)	0.0624 (3)	0.128 (2)	
H13A	−0.199280	0.193234	0.100073	0.192*	
H13B	−0.227740	0.150391	0.034807	0.192*	
H13C	−0.353785	0.218854	0.073176	0.192*	
C14	0.3028 (3)	0.1878 (2)	0.12996 (13)	0.0426 (6)	
C15	0.3031 (4)	0.1708 (2)	0.06598 (16)	0.0604 (8)	
H15	0.287552	0.224799	0.038894	0.073*	
C16	0.3263 (5)	0.0746 (3)	0.04233 (19)	0.0793 (12)	
H16	0.325846	0.064160	−0.000539	0.095*	
C17	0.3502 (5)	−0.0066 (3)	0.0820 (2)	0.0773 (11)	
H17	0.365477	−0.071499	0.065966	0.093*	
C18	0.3512 (4)	0.0093 (2)	0.14495 (19)	0.0652 (9)	
H18	0.368046	−0.044899	0.171690	0.078*	
C19	0.3273 (3)	0.1054 (2)	0.16911 (15)	0.0509 (7)	
H19	0.327561	0.115159	0.212065	0.061*	
C20	0.5421 (3)	0.45903 (19)	0.24069 (11)	0.0351 (5)	
C21	0.6551 (3)	0.3940 (2)	0.25902 (14)	0.0480 (7)	
H21	0.651441	0.324875	0.252079	0.058*	
C22	0.7730 (3)	0.4332 (3)	0.28766 (16)	0.0555 (8)	
H22	0.849573	0.390617	0.300053	0.067*	
C23	0.7765 (3)	0.5349 (2)	0.29771 (15)	0.0519 (7)	
H23	0.855142	0.562503	0.316873	0.062*	
C24	0.6613 (3)	0.5953 (2)	0.27886 (14)	0.0470 (6)	
H24	0.663814	0.664509	0.285689	0.056*	
C25	0.3019 (3)	0.75430 (18)	0.46657 (11)	0.0312 (5)	
C26	0.4121 (3)	0.6987 (2)	0.42594 (12)	0.0388 (6)	
H26	0.404769	0.697817	0.382954	0.047*	
C27	0.5282 (3)	0.6468 (2)	0.44870 (12)	0.0435 (6)	
H27	0.599206	0.611244	0.420730	0.052*	
C28	0.5450 (3)	0.6448 (2)	0.51415 (12)	0.0402 (6)	
C29	0.4400 (3)	0.70200 (19)	0.55394 (11)	0.0361 (5)	
H29	0.450440	0.703967	0.596623	0.043*	
C30	0.3186 (3)	0.75706 (17)	0.53179 (11)	0.0314 (5)	
C31	0.2053 (3)	0.81444 (18)	0.57144 (11)	0.0313 (5)	
C32	0.0798 (3)	0.85298 (18)	0.54550 (11)	0.0336 (5)	
H32	0.001934	0.887326	0.570944	0.040*	
C33	0.0682 (3)	0.84097 (17)	0.48098 (11)	0.0318 (5)	
C34A	0.7695 (5)	0.5291 (3)	0.49221 (17)	0.0598 (4)	0.878 (4)
H34A	0.716880	0.495813	0.461332	0.072*	0.878 (4)
H34B	0.819456	0.477394	0.515525	0.072*	0.878 (4)
C35A	0.8880 (5)	0.5987 (3)	0.45924 (17)	0.0598 (4)	0.878 (4)
H35A	0.837950	0.651735	0.437884	0.090*	0.878 (4)
H35B	0.957178	0.561486	0.429056	0.090*	0.878 (4)
H35C	0.945156	0.627778	0.489612	0.090*	0.878 (4)
C34B	0.8079 (18)	0.5859 (16)	0.4945 (10)	0.0598 (4)	0.122 (4)
H34C	0.890775	0.589382	0.520309	0.072*	0.122 (4)
H34D	0.813426	0.644931	0.466687	0.072*	0.122 (4)
C35B	0.826 (3)	0.4903 (17)	0.4557 (9)	0.0598 (4)	0.122 (4)
H35D	0.828382	0.432266	0.483290	0.090*	0.122 (4)
H35E	0.920930	0.492781	0.427229	0.090*	0.122 (4)
H35F	0.741117	0.485305	0.431911	0.090*	0.122 (4)
C36	0.6734 (4)	0.5767 (2)	0.60222 (14)	0.0530 (7)	
H36A	0.722257	0.511940	0.609811	0.064*	
H36B	0.571034	0.576515	0.626112	0.064*	
C37	0.7654 (5)	0.6594 (3)	0.62720 (18)	0.0799 (11)	
H37A	0.863950	0.664467	0.601628	0.120*	
H37B	0.779985	0.643394	0.670001	0.120*	
H37C	0.710372	0.722741	0.625797	0.120*	
C38	0.2191 (3)	0.83535 (18)	0.63866 (11)	0.0345 (5)	
C39	0.3567 (3)	0.8713 (2)	0.65593 (12)	0.0405 (6)	
H39	0.443295	0.876552	0.625739	0.049*	
C40	0.3653 (4)	0.8991 (2)	0.71739 (14)	0.0514 (7)	
H40	0.456951	0.924070	0.728097	0.062*	
C41	0.2390 (4)	0.8900 (3)	0.76260 (14)	0.0613 (9)	
H41	0.245125	0.908451	0.803944	0.074*	
C42	0.1030 (4)	0.8535 (3)	0.74660 (14)	0.0589 (8)	
H42	0.017851	0.846313	0.777370	0.071*	
C43	0.0927 (3)	0.8274 (2)	0.68500 (13)	0.0456 (6)	
H43	−0.000234	0.804239	0.674505	0.055*	
C44	−0.0744 (3)	0.87005 (18)	0.45391 (12)	0.0338 (5)	
C45	−0.2125 (3)	0.8899 (2)	0.49184 (13)	0.0416 (6)	
H45	−0.215800	0.893448	0.535407	0.050*	
C46	−0.3456 (3)	0.9044 (2)	0.46411 (15)	0.0480 (7)	
H46	−0.439540	0.916770	0.488922	0.058*	
C47	−0.3376 (3)	0.9002 (2)	0.39932 (15)	0.0495 (7)	
H47	−0.425572	0.909121	0.379625	0.059*	
C48	−0.1953 (3)	0.8826 (2)	0.36457 (14)	0.0486 (7)	
H48	−0.189055	0.880983	0.320833	0.058*	

### Aqua-1κO-di-µ-chlorido-1:2κ4Cl:Cl-dichlorido-1κCl,2κCl-bis[6-(diethylamino)-4-phenyl-2-(pyridin-2-yl)quinoline]-1κ2N1,N2;2κ2N1,N2-dimanganese(II) Atomic displacement parameters (Å^2^)

**Table:**

	*U*^11^	*U*^22^	*U*^33^	*U*^12^	*U*^13^	*U*^23^
Mn1	0.0468 (2)	0.0371 (2)	0.0362 (2)	0.00307 (17)	−0.01036 (17)	−0.00673 (16)
Cl1A	0.0741 (13)	0.0631 (8)	0.0429 (6)	0.0085 (8)	−0.0122 (7)	0.0016 (5)
Cl1B	0.0741 (13)	0.0631 (8)	0.0429 (6)	0.0085 (8)	−0.0122 (7)	0.0016 (5)
O1	0.0712 (16)	0.0835 (19)	0.0555 (14)	0.0005 (14)	−0.0230 (12)	0.0105 (13)
N1	0.0404 (11)	0.0364 (11)	0.0340 (10)	0.0031 (9)	−0.0089 (9)	−0.0066 (8)
C1	0.0423 (14)	0.0410 (14)	0.0331 (12)	0.0003 (11)	−0.0100 (10)	−0.0046 (10)
Mn2	0.0388 (2)	0.0400 (2)	0.0325 (2)	0.00306 (16)	−0.00725 (15)	−0.00383 (15)
Cl2	0.0467 (4)	0.0396 (3)	0.0439 (3)	−0.0031 (3)	−0.0039 (3)	−0.0069 (3)
N2	0.0637 (17)	0.0703 (19)	0.0751 (19)	0.0021 (14)	−0.0430 (15)	−0.0104 (14)
C2	0.0512 (16)	0.0470 (16)	0.0492 (16)	0.0089 (13)	−0.0165 (13)	−0.0126 (13)
Cl3	0.0507 (4)	0.0526 (4)	0.0563 (4)	−0.0139 (3)	0.0052 (3)	−0.0198 (3)
N3	0.0409 (12)	0.0381 (12)	0.0386 (11)	−0.0021 (9)	−0.0085 (9)	−0.0060 (9)
C3	0.0471 (16)	0.0583 (18)	0.0496 (16)	0.0115 (13)	−0.0185 (13)	−0.0098 (13)
Cl4	0.0548 (4)	0.0441 (4)	0.0592 (4)	−0.0025 (3)	0.0015 (3)	−0.0111 (3)
N4	0.0322 (10)	0.0324 (10)	0.0304 (10)	0.0026 (8)	−0.0067 (8)	−0.0021 (8)
C4	0.0497 (16)	0.0528 (17)	0.0427 (15)	−0.0033 (13)	−0.0154 (12)	−0.0023 (12)
N5	0.0552 (15)	0.0649 (16)	0.0421 (13)	0.0296 (12)	−0.0104 (11)	−0.0019 (11)
C5	0.0550 (17)	0.0411 (15)	0.0457 (15)	−0.0044 (12)	−0.0185 (13)	−0.0030 (12)
N6	0.0354 (11)	0.0375 (11)	0.0412 (12)	0.0032 (9)	−0.0121 (9)	−0.0008 (9)
C6	0.0451 (14)	0.0363 (13)	0.0331 (12)	−0.0010 (11)	−0.0083 (10)	−0.0004 (10)
C7	0.0478 (15)	0.0351 (13)	0.0354 (13)	−0.0021 (11)	−0.0089 (11)	0.0007 (10)
C8	0.0441 (14)	0.0345 (13)	0.0405 (13)	0.0018 (11)	−0.0110 (11)	0.0000 (10)
C9	0.0411 (13)	0.0367 (13)	0.0308 (12)	−0.0019 (10)	−0.0068 (10)	−0.0013 (10)
C10	0.0467 (17)	0.077 (2)	0.0538 (18)	−0.0056 (15)	−0.0180 (14)	0.0053 (16)
C11	0.088 (3)	0.102 (3)	0.060 (2)	−0.006 (2)	−0.014 (2)	0.018 (2)
C12	0.088 (3)	0.093 (3)	0.087 (3)	−0.010 (2)	−0.054 (2)	−0.003 (2)
C13	0.131 (5)	0.113 (4)	0.147 (5)	−0.035 (4)	−0.039 (4)	0.000 (4)
C14	0.0468 (15)	0.0362 (14)	0.0470 (15)	−0.0014 (11)	−0.0146 (12)	−0.0023 (11)
C15	0.089 (2)	0.0436 (17)	0.0519 (17)	0.0053 (16)	−0.0220 (16)	−0.0061 (13)
C16	0.121 (3)	0.057 (2)	0.065 (2)	0.010 (2)	−0.030 (2)	−0.0248 (18)
C17	0.099 (3)	0.0407 (18)	0.095 (3)	0.0075 (18)	−0.019 (2)	−0.0166 (18)
C18	0.071 (2)	0.0390 (17)	0.087 (3)	0.0017 (15)	−0.0154 (19)	0.0088 (16)
C19	0.0550 (17)	0.0434 (16)	0.0563 (17)	−0.0001 (13)	−0.0156 (14)	0.0044 (13)
C20	0.0344 (12)	0.0385 (13)	0.0320 (12)	−0.0027 (10)	−0.0028 (9)	−0.0015 (10)
C21	0.0484 (16)	0.0419 (15)	0.0567 (17)	0.0026 (12)	−0.0178 (13)	−0.0021 (13)
C22	0.0441 (16)	0.0583 (19)	0.068 (2)	0.0048 (14)	−0.0215 (14)	−0.0008 (15)
C23	0.0396 (15)	0.0601 (19)	0.0588 (18)	−0.0070 (13)	−0.0156 (13)	−0.0073 (14)
C24	0.0423 (15)	0.0476 (16)	0.0530 (16)	−0.0033 (12)	−0.0116 (12)	−0.0087 (13)
C25	0.0295 (11)	0.0319 (12)	0.0323 (12)	0.0025 (9)	−0.0050 (9)	−0.0002 (9)
C26	0.0406 (14)	0.0460 (15)	0.0296 (12)	0.0077 (11)	−0.0052 (10)	−0.0033 (10)
C27	0.0388 (14)	0.0531 (16)	0.0383 (14)	0.0148 (12)	−0.0051 (11)	−0.0078 (12)
C28	0.0374 (13)	0.0424 (15)	0.0410 (14)	0.0091 (11)	−0.0078 (11)	−0.0003 (11)
C29	0.0380 (13)	0.0403 (14)	0.0303 (12)	0.0075 (10)	−0.0069 (10)	−0.0001 (10)
C30	0.0311 (11)	0.0303 (12)	0.0329 (12)	0.0011 (9)	−0.0045 (9)	0.0002 (9)
C31	0.0313 (12)	0.0308 (12)	0.0317 (11)	−0.0003 (9)	−0.0039 (9)	−0.0013 (9)
C32	0.0315 (12)	0.0345 (13)	0.0344 (12)	0.0051 (10)	−0.0031 (9)	−0.0053 (10)
C33	0.0314 (12)	0.0298 (12)	0.0346 (12)	0.0024 (9)	−0.0061 (9)	−0.0012 (9)
C34A	0.0741 (13)	0.0631 (8)	0.0429 (6)	0.0085 (8)	−0.0122 (7)	0.0016 (5)
C35A	0.0741 (13)	0.0631 (8)	0.0429 (6)	0.0085 (8)	−0.0122 (7)	0.0016 (5)
C34B	0.0741 (13)	0.0631 (8)	0.0429 (6)	0.0085 (8)	−0.0122 (7)	0.0016 (5)
C35B	0.0741 (13)	0.0631 (8)	0.0429 (6)	0.0085 (8)	−0.0122 (7)	0.0016 (5)
C36	0.0552 (17)	0.0551 (18)	0.0505 (17)	0.0177 (14)	−0.0166 (13)	0.0058 (13)
C37	0.083 (3)	0.098 (3)	0.061 (2)	−0.013 (2)	−0.0170 (19)	−0.004 (2)
C38	0.0393 (13)	0.0320 (12)	0.0321 (12)	0.0066 (10)	−0.0055 (10)	−0.0023 (9)
C39	0.0430 (14)	0.0402 (14)	0.0391 (13)	0.0027 (11)	−0.0088 (11)	−0.0007 (11)
C40	0.0636 (19)	0.0488 (17)	0.0466 (16)	0.0016 (14)	−0.0248 (14)	−0.0054 (13)
C41	0.093 (3)	0.0584 (19)	0.0349 (15)	0.0158 (18)	−0.0183 (16)	−0.0083 (13)
C42	0.064 (2)	0.070 (2)	0.0378 (15)	0.0162 (16)	0.0062 (14)	−0.0025 (14)
C43	0.0440 (15)	0.0521 (17)	0.0399 (14)	0.0035 (12)	−0.0023 (11)	−0.0013 (12)
C44	0.0326 (12)	0.0291 (12)	0.0407 (13)	0.0011 (9)	−0.0089 (10)	−0.0025 (10)
C45	0.0365 (13)	0.0435 (15)	0.0446 (14)	0.0064 (11)	−0.0055 (11)	−0.0028 (11)
C46	0.0317 (13)	0.0477 (16)	0.0632 (18)	0.0058 (11)	−0.0037 (12)	0.0046 (13)
C47	0.0352 (14)	0.0475 (16)	0.069 (2)	0.0007 (12)	−0.0213 (13)	0.0081 (14)
C48	0.0457 (16)	0.0544 (17)	0.0487 (16)	0.0004 (13)	−0.0179 (13)	0.0010 (13)

### Aqua-1κO-di-µ-chlorido-1:2κ4Cl:Cl-dichlorido-1κCl,2κCl-bis[6-(diethylamino)-4-phenyl-2-(pyridin-2-yl)quinoline]-1κ2N1,N2;2κ2N1,N2-dimanganese(II) Geometric parameters (Å, º)

**Table:**

Mn1—Cl1A	2.382 (2)	C12—C13	1.437 (7)
Mn1—Cl1B	2.337 (4)	C13—H13A	0.9600
Mn1—N1	2.286 (2)	C13—H13B	0.9600
Mn1—Cl2	2.4974 (7)	C13—H13C	0.9600
Mn1—Cl3	2.4501 (8)	C14—C15	1.390 (4)
Mn1—N3	2.215 (2)	C14—C19	1.392 (4)
C34Aa—H34A	0.9700	C15—H15	0.9300
C34Aa—H34B	0.9700	C15—C16	1.378 (5)
C34Aa—C35A	1.494 (6)	C16—H16	0.9300
C35Aa—H35A	0.9600	C16—C17	1.387 (5)
C35Aa—H35B	0.9600	C17—H17	0.9300
C35Aa—H35C	0.9600	C17—C18	1.367 (5)
C34Bb—H34C	0.9700	C18—H18	0.9300
C34Bb—H34D	0.9700	C18—C19	1.382 (5)
C34Bb—C35B	1.520 (10)	C19—H19	0.9300
C35Bb—H35D	0.9600	C20—C21	1.390 (4)
C35Bb—H35E	0.9600	C21—H21	0.9300
C35Bb—H35F	0.9600	C21—C22	1.381 (4)
O1—H1A	0.816 (10)	C22—H22	0.9300
O1—H1B	0.832 (10)	C22—C23	1.366 (4)
O1—Mn2	2.213 (2)	C23—H23	0.9300
N1—C1	1.364 (3)	C23—C24	1.373 (4)
N1—C9	1.328 (3)	C24—H24	0.9300
C1—C2	1.418 (4)	C25—C26	1.413 (3)
C1—C6	1.418 (4)	C25—C30	1.423 (3)
Mn2—Cl2	2.6269 (8)	C26—H26	0.9300
Mn2—Cl3	2.5838 (8)	C26—C27	1.354 (4)
Mn2—Cl4	2.4354 (8)	C27—H27	0.9300
Mn2—N4	2.3087 (19)	C27—C28	1.426 (4)
Mn2—N6	2.257 (2)	C28—C29	1.389 (4)
N2—C4	1.383 (4)	C29—H29	0.9300
N2—C10	1.462 (4)	C29—C30	1.406 (3)
N2—C12	1.521 (5)	C30—C31	1.432 (3)
C2—H2	0.9300	C31—C32	1.380 (3)
C2—C3	1.360 (4)	C31—C38	1.490 (3)
N3—C20	1.348 (3)	C32—H32	0.9300
N3—C24	1.346 (3)	C32—C33	1.409 (3)
C3—H3	0.9300	C33—C44	1.485 (3)
C3—C4	1.415 (4)	C36—H36A	0.9700
N4—C25	1.371 (3)	C36—H36B	0.9700
N4—C33	1.325 (3)	C36—C37	1.515 (5)
C4—C5	1.387 (4)	C37—H37A	0.9600
N5—C28	1.370 (3)	C37—H37B	0.9600
N5—C34A	1.455 (4)	C37—H37C	0.9600
N5—C34B	1.474 (10)	C38—C39	1.398 (4)
N5—C36	1.456 (4)	C38—C43	1.387 (4)
C5—H5	0.9300	C39—H39	0.9300
C5—C6	1.415 (4)	C39—C40	1.383 (4)
N6—C44	1.345 (3)	C40—H40	0.9300
N6—C48	1.339 (3)	C40—C41	1.373 (5)
C6—C7	1.431 (4)	C41—H41	0.9300
C7—C8	1.367 (4)	C41—C42	1.379 (5)
C7—C14	1.489 (4)	C42—H42	0.9300
C8—H8	0.9300	C42—C43	1.383 (4)
C8—C9	1.403 (4)	C43—H43	0.9300
C9—C20	1.487 (3)	C44—C45	1.386 (3)
C10—H10A	0.9700	C45—H45	0.9300
C10—H10B	0.9700	C45—C46	1.385 (4)
C10—C11	1.502 (5)	C46—H46	0.9300
C11—H11A	0.9600	C46—C47	1.380 (4)
C11—H11B	0.9600	C47—H47	0.9300
C11—H11C	0.9600	C47—C48	1.380 (4)
C12—H12A	0.9700	C48—H48	0.9300
C12—H12B	0.9700		
			
CL1Aa—Mn1—Cl2	99.12 (6)	N2—C12—H12A	109.4
N1—Mn1—Cl1A	92.70 (8)	N2—C12—H12B	109.4
CL1Bb—Mn1—Cl2	97.93 (15)	H12A—C12—H12B	108.0
CL1Aa—Mn1—Cl3	132.16 (10)	C13—C12—N2	111.0 (4)
N1—Mn1—Cl1B	95.38 (16)	C13—C12—H12A	109.4
N1—Mn1—Cl2	165.37 (6)	C13—C12—H12B	109.4
N1—Mn1—Cl3	92.21 (6)	C12—C13—H13A	109.5
Cl3—Mn1—Cl2	86.25 (2)	C12—C13—H13B	109.5
N3—Mn1—Cl1A	113.64 (12)	C12—C13—H13C	109.5
N3—Mn1—Cl1B	125.41 (18)	H13A—C13—H13B	109.5
N3—Mn1—N1	73.33 (8)	H13A—C13—H13C	109.5
N3—Mn1—Cl2	93.91 (6)	H13B—C13—H13C	109.5
N3—Mn1—Cl3	113.33 (6)	C15—C14—C7	121.4 (2)
CL1Bb—Mn1—Cl3	120.46 (18)	C15—C14—C19	118.2 (3)
H1A—O1—H1B	111 (5)	C19—C14—C7	120.0 (2)
C35Aa—C34Aa—H34A	109.6	C14—C15—H15	119.7
H34Aa—C34Aa—H34B	108.1	C16—C15—C14	120.6 (3)
Mn2—O1—H1A	110 (4)	C16—C15—H15	119.7
C35Aa—C34Aa—H34B	109.6	C15—C16—H16	119.8
C34Aa—C35Aa—H35A	109.5	C15—C16—C17	120.4 (4)
H35Aa—C35Aa—H35B	109.5	C17—C16—H16	119.8
C34Aa—C35Aa—H35B	109.5	C16—C17—H17	120.3
H35Ba—C35Aa—H35C	109.5	C18—C17—C16	119.5 (3)
H35Aa—C35Aa—H35C	109.5	C18—C17—H17	120.3
Mn2—O1—H1B	125 (3)	C17—C18—H18	119.8
C1—N1—Mn1	125.31 (17)	C17—C18—C19	120.5 (3)
C9—N1—Mn1	115.29 (16)	C19—C18—H18	119.8
C9—N1—C1	118.7 (2)	C14—C19—H19	119.6
N1—C1—C2	119.2 (2)	C18—C19—C14	120.8 (3)
N1—C1—C6	123.0 (2)	C18—C19—H19	119.6
C6—C1—C2	117.8 (2)	N3—C20—C9	116.1 (2)
O1—Mn2—Cl2	91.02 (8)	N3—C20—C21	121.2 (2)
O1—Mn2—Cl3	88.16 (8)	C21—C20—C9	122.8 (2)
O1—Mn2—Cl4	87.81 (8)	C20—C21—H21	120.4
O1—Mn2—N4	158.31 (9)	C22—C21—C20	119.2 (3)
O1—Mn2—N6	86.49 (9)	C22—C21—H21	120.4
Cl3—Mn2—Cl2	80.95 (2)	C21—C22—H22	120.2
Cl4—Mn2—Cl2	89.05 (3)	C23—C22—C21	119.7 (3)
Cl4—Mn2—Cl3	169.15 (3)	C23—C22—H22	120.2
N4—Mn2—Cl2	110.53 (5)	C22—C23—H23	120.8
N4—Mn2—Cl3	92.69 (5)	C22—C23—C24	118.5 (3)
N4—Mn2—Cl4	94.77 (5)	C24—C23—H23	120.8
N6—Mn2—Cl2	175.19 (6)	N3—C24—C23	123.2 (3)
N6—Mn2—Cl3	94.86 (6)	N3—C24—H24	118.4
N6—Mn2—Cl4	94.95 (6)	C23—C24—H24	118.4
N6—Mn2—N4	71.83 (7)	N4—C25—C26	118.3 (2)
Mn1—Cl2—Mn2	93.75 (2)	N4—C25—C30	123.3 (2)
C34Aa—C35Aa—H35C	109.5	C26—C25—C30	118.4 (2)
C35Bb—C34Bb—H34C	109.7	C25—C26—H26	119.5
H34Cb—C34Bb—H34D	108.2	C27—C26—C25	120.9 (2)
C35Bb—C34Bb—H34D	109.7	C27—C26—H26	119.5
C34Bb—C35Bb—H35D	109.5	C26—C27—H27	118.9
H35Db—C35Bb—H35E	109.5	C26—C27—C28	122.1 (2)
C34Bb—C35Bb—H35E	109.5	C28—C27—H27	118.9
H35Db—C35Bb—H35F	109.5	N5—C28—C27	119.8 (2)
C34Bb—C35Bb—H35F	109.5	N5—C28—C29	123.0 (2)
H35Eb—C35Bb—H35F	109.5	C29—C28—C27	117.1 (2)
C34Aa—N5—C36	116.7 (2)	C28—C29—H29	118.9
C4—N2—C10	122.0 (3)	C28—C29—C30	122.1 (2)
C4—N2—C12	119.2 (3)	C30—C29—H29	118.9
C10—N2—C12	116.8 (3)	C25—C30—C31	116.8 (2)
C1—C2—H2	119.3	C29—C30—C25	119.2 (2)
C3—C2—C1	121.5 (3)	C29—C30—C31	124.0 (2)
C3—C2—H2	119.3	C30—C31—C38	122.7 (2)
Mn1—Cl3—Mn2	95.97 (3)	C32—C31—C30	118.1 (2)
C20—N3—Mn1	117.72 (16)	C32—C31—C38	119.2 (2)
C24—N3—Mn1	124.00 (19)	C31—C32—H32	119.6
C24—N3—C20	118.3 (2)	C31—C32—C33	120.9 (2)
C2—C3—H3	119.1	C33—C32—H32	119.6
C2—C3—C4	121.8 (3)	N4—C33—C32	122.1 (2)
C4—C3—H3	119.1	N4—C33—C44	116.2 (2)
C25—N4—Mn2	126.91 (15)	C32—C33—C44	121.6 (2)
C33—N4—Mn2	113.09 (15)	N5—C36—H36A	108.5
C33—N4—C25	118.2 (2)	N5—C36—H36B	108.5
N2—C4—C3	120.9 (3)	N5—C36—C37	115.2 (3)
N2—C4—C5	121.6 (3)	H36A—C36—H36B	107.5
C5—C4—C3	117.6 (3)	C37—C36—H36A	108.5
N5—C34Aa—H34A	109.6	C37—C36—H36B	108.5
N5—C34Aa—H34B	109.6	C36—C37—H37A	109.5
N5—C34Aa—C35A	110.2 (3)	C36—C37—H37B	109.5
C28—N5—C34A	121.7 (2)	C36—C37—H37C	109.5
C28—N5—C34B	115.0 (11)	H37A—C37—H37B	109.5
C28—N5—C36	121.6 (2)	H37A—C37—H37C	109.5
C36—N5—C34B	112.9 (10)	H37B—C37—H37C	109.5
C4—C5—H5	119.1	C39—C38—C31	120.8 (2)
C4—C5—C6	121.9 (3)	C43—C38—C31	120.9 (2)
C6—C5—H5	119.1	C43—C38—C39	118.1 (2)
C44—N6—Mn2	115.72 (15)	C38—C39—H39	119.6
C48—N6—Mn2	125.71 (19)	C40—C39—C38	120.8 (3)
C48—N6—C44	118.5 (2)	C40—C39—H39	119.6
C1—C6—C7	117.1 (2)	C39—C40—H40	119.9
C5—C6—C1	119.4 (2)	C41—C40—C39	120.1 (3)
C5—C6—C7	123.5 (2)	C41—C40—H40	119.9
C6—C7—C14	123.1 (2)	C40—C41—H41	120.1
C8—C7—C6	117.9 (2)	C40—C41—C42	119.9 (3)
C8—C7—C14	119.0 (2)	C42—C41—H41	120.1
C7—C8—H8	119.2	C41—C42—H42	119.9
C7—C8—C9	121.6 (2)	C41—C42—C43	120.3 (3)
C9—C8—H8	119.2	C43—C42—H42	119.9
N1—C9—C8	121.6 (2)	C38—C43—H43	119.6
N1—C9—C20	117.1 (2)	C42—C43—C38	120.8 (3)
C8—C9—C20	121.3 (2)	C42—C43—H43	119.6
N2—C10—H10A	108.8	N6—C44—C33	116.4 (2)
N2—C10—H10B	108.8	N6—C44—C45	121.5 (2)
N2—C10—C11	113.6 (3)	C45—C44—C33	121.8 (2)
H10A—C10—H10B	107.7	C44—C45—H45	120.4
C11—C10—H10A	108.8	C46—C45—C44	119.2 (3)
C11—C10—H10B	108.8	C46—C45—H45	120.4
C10—C11—H11A	109.5	C45—C46—H46	120.3
C10—C11—H11B	109.5	C47—C46—C45	119.4 (3)
C10—C11—H11C	109.5	C47—C46—H46	120.3
H11A—C11—H11B	109.5	C46—C47—H47	121.0
H11A—C11—H11C	109.5	C46—C47—C48	118.1 (3)
N5—C34Bb—H34C	109.7	C48—C47—H47	121.0
N5—C34Bb—H34D	109.7	N6—C48—C47	123.3 (3)
N5—C34Bb—C35B	109.7 (10)	N6—C48—H48	118.4
H11B—C11—H11C	109.5	C47—C48—H48	118.4
			
Mn1—N1—C1—C2	−12.0 (3)	C15—C14—C19—C18	0.0 (5)
Mn1—N1—C1—C6	167.64 (18)	C15—C16—C17—C18	−0.2 (7)
Mn1—N1—C9—C8	−169.87 (18)	C16—C17—C18—C19	0.5 (6)
Mn1—N1—C9—C20	8.1 (3)	C17—C18—C19—C14	−0.4 (5)
Mn1—N3—C20—C9	2.2 (3)	C19—C14—C15—C16	0.3 (5)
Mn1—N3—C20—C21	−177.6 (2)	C20—N3—C24—C23	−0.5 (4)
Mn1—N3—C24—C23	177.8 (2)	C20—C21—C22—C23	0.2 (5)
N1—C1—C2—C3	178.9 (3)	C21—C22—C23—C24	0.1 (5)
N1—C1—C6—C5	−179.8 (2)	C22—C23—C24—N3	0.0 (5)
N1—C1—C6—C7	0.6 (4)	C24—N3—C20—C9	−179.3 (2)
N1—C9—C20—N3	−7.1 (3)	C24—N3—C20—C21	0.8 (4)
N1—C9—C20—C21	172.8 (2)	C25—N4—C33—C32	8.8 (3)
C1—N1—C9—C8	1.0 (4)	C25—N4—C33—C44	−168.2 (2)
C1—N1—C9—C20	179.0 (2)	C25—C26—C27—C28	0.4 (4)
C1—C2—C3—C4	−0.2 (5)	C25—C30—C31—C32	7.1 (3)
C1—C6—C7—C8	2.2 (4)	C25—C30—C31—C38	−171.8 (2)
C1—C6—C7—C14	−176.5 (2)	C26—C25—C30—C29	−2.2 (3)
Mn2—N4—C25—C26	−24.3 (3)	C26—C25—C30—C31	179.7 (2)
Mn2—N4—C25—C30	158.73 (17)	C26—C27—C28—N5	175.5 (3)
Mn2—N4—C33—C32	−156.83 (18)	C26—C27—C28—C29	−2.5 (4)
Mn2—N4—C33—C44	26.1 (2)	C27—C28—C29—C30	2.2 (4)
Mn2—N6—C44—C33	−10.3 (3)	C28—N5—C34Bb—C35Bb	−101.4 (18)
Mn2—N6—C44—C45	175.29 (19)	C28—N5—C34Aa—C35Aa	75.9 (4)
Mn2—N6—C48—C47	−176.6 (2)	C28—N5—C36—C37	−84.6 (4)
N2—C4—C5—C6	177.2 (3)	C28—C29—C30—C25	0.1 (4)
C2—C1—C6—C5	−0.1 (4)	C28—C29—C30—C31	178.0 (2)
C2—C1—C6—C7	−179.7 (2)	C29—C30—C31—C32	−170.8 (2)
C2—C3—C4—N2	−178.1 (3)	C29—C30—C31—C38	10.3 (4)
C2—C3—C4—C5	2.0 (5)	C30—C25—C26—C27	2.0 (4)
N3—C20—C21—C22	−0.7 (4)	C30—C31—C32—C33	−3.5 (3)
C3—C4—C5—C6	−2.9 (4)	C30—C31—C38—C39	48.1 (3)
N4—C25—C26—C27	−175.1 (2)	C30—C31—C38—C43	−137.0 (3)
N4—C25—C30—C29	174.8 (2)	C31—C32—C33—N4	−4.8 (4)
N4—C25—C30—C31	−3.3 (3)	C31—C32—C33—C44	172.0 (2)
N4—C33—C44—N6	−11.2 (3)	C31—C38—C39—C40	174.3 (2)
N4—C33—C44—C45	163.2 (2)	C31—C38—C43—C42	−175.6 (3)
C4—N2—C10—C11	85.2 (4)	C32—C31—C38—C39	−130.8 (3)
C4—N2—C12—C13	96.5 (5)	C32—C31—C38—C43	44.1 (3)
C4—C5—C6—C1	2.0 (4)	C32—C33—C44—N6	171.7 (2)
C4—C5—C6—C7	−178.4 (3)	C32—C33—C44—C45	−13.9 (4)
N5—C28—C29—C30	−175.7 (3)	C33—N4—C25—C26	172.3 (2)
C5—C6—C7—C8	−177.4 (3)	C33—N4—C25—C30	−4.7 (3)
C5—C6—C7—C14	3.9 (4)	C34Aa—N5—C28—C27	4.3 (5)
N6—C44—C45—C46	2.1 (4)	C34Bb—N5—C28—C27	41.2 (9)
C6—C1—C2—C3	−0.8 (4)	C34Aa—N5—C28—C29	−177.9 (3)
C6—C7—C8—C9	−3.4 (4)	C34Bb—N5—C28—C29	−140.9 (8)
C6—C7—C14—C15	56.2 (4)	C33—C44—C45—C46	−172.1 (2)
C6—C7—C14—C19	−130.4 (3)	C36—N5—C34Bb—C35Bb	112.9 (17)
C7—C8—C9—N1	1.9 (4)	C36—N5—C34Aa—C35Aa	−103.5 (3)
C7—C8—C9—C20	−176.0 (2)	C36—N5—C28—C27	−176.4 (3)
C7—C14—C15—C16	173.8 (3)	C36—N5—C28—C29	1.5 (5)
C7—C14—C19—C18	−173.5 (3)	C38—C31—C32—C33	175.4 (2)
C8—C7—C14—C15	−122.5 (3)	C34Aa—N5—C36—C37	94.8 (4)
C8—C7—C14—C19	50.9 (4)	C34Bb—N5—C36—C37	58.5 (9)
C8—C9—C20—N3	170.9 (2)	C38—C39—C40—C41	1.2 (4)
C8—C9—C20—C21	−9.2 (4)	C39—C38—C43—C42	−0.6 (4)
C9—N1—C1—C2	178.1 (2)	C39—C40—C41—C42	−0.3 (5)
C9—N1—C1—C6	−2.2 (4)	C40—C41—C42—C43	−1.0 (5)
C9—C20—C21—C22	179.4 (3)	C41—C42—C43—C38	1.4 (5)
C10—N2—C4—C3	−3.1 (5)	C43—C38—C39—C40	−0.7 (4)
C10—N2—C4—C5	176.8 (3)	C44—N6—C48—C47	−0.1 (4)
C10—N2—C12—C13	−99.2 (4)	C44—C45—C46—C47	−1.0 (4)
C12—N2—C4—C3	160.4 (3)	C45—C46—C47—C48	−0.6 (4)
C12—N2—C4—C5	−19.8 (5)	C46—C47—C48—N6	1.2 (5)
C12—N2—C10—C11	−78.6 (4)	C48—N6—C44—C33	172.9 (2)
C14—C7—C8—C9	175.3 (2)	C48—N6—C44—C45	−1.5 (4)
C14—C15—C16—C17	−0.2 (6)		

### Aqua-1κO-di-µ-chlorido-1:2κ4Cl:Cl-dichlorido-1κCl,2κCl-bis[6-(diethylamino)-4-phenyl-2-(pyridin-2-yl)quinoline]-1κ2N1,N2;2κ2N1,N2-dimanganese(II) Hydrogen-bond geometry (Å, º)

*Cg1*–*Cg*4 are the centroids of the C1–C6, C38–C43, N3/C20–C24 and C25–C30 rings, respectively.

**Table:**

*D*—H···*A*	*D*—H	H···*A*	*D*···*A*	*D*—H···*A*
C12—H12*A*···Cl1*B*^i^	0.97	2.81	3.734 (7)	158
C23—H23···Cl3^ii^	0.93	2.74	3.567 (3)	149
C45—H45···Cl4^iii^	0.93	2.69	3.563 (3)	157
C11—H11*A*···*Cg*1^i^	0.96	2.97	3.585 (4)	123
C21—H21···*Cg*2^iv^	0.93	2.89	3.642 (3)	139
C36—H36*B*···*Cg*3^iv^	0.97	2.96	3.845 (3)	153
C35*B*—H35*D*···*Cg*4^iv^	0.96	2.84	3.45 (2)	122

Symmetry codes: (i) −*x*, −*y*+1, −*z*; (ii) *x*+1, *y*, *z*; (iii) −*x*, −*y*+2, −*z*+1; (iv) −*x*+1, −*y*+1, −*z*+1.
