# Supplementary material for: Synthesis, crystal structure and photophysical properties of a dinuclear MnII complex with 6-(di­ethyl­amino)-4-phenyl-2-(pyridin-2-yl)quinoline
Source: Acta Crystallogr E Crystallogr Commun. 2024 Jun 28;80(Pt 7):795–9. doi: 10.1107/S2056989024006042 (PMC11223701; doi:10.1107/S2056989024006042)
Supplement: Supplementary file 3 [file e-80-00795-sup3.pdf]

# SYNTHESIS, CRYSTAL STRUCTURE AND PHOTOPHYSICAL PROPERTIES OF THE DINUCLEAR Mn(II) COMPLEX WITH 6-(*N,N*-DIETHYLAMINE)-4-PHENYL-2(PYRIDIN-2-YL)QUINOLINE

**Le Thi Hong Hai[1,2], Hoang Tuan Duong[1], Nguyen Duc Anh[1], Nguyen Hien[1] and Luc Van Meervelt[3]**

[1]*Faculty of Chemistry, Hanoi National University of Education, Hanoi, Vietnam*
[2] *Institute of Natural Sciences, Hanoi National University of Education, Hanoi, Vietnam*
[3]*Department of Chemistry, KU Leuven, Celestijnenlaan 200F, B-3001 Leuven, Belgium*

## Supporting Information

**Figure S1.** ESI-MS spectra of **QP**.

**Figure S2.** FT-IR spectra of **QP**.

**Figure S3.** $^1$H NMR spectra of **QP** in $CDCl_3$.

**Figure S4.** ESI-MS spectra of **MnQP**.

**Figure S5.** FT-IR spectra of **MnQP**.

**Figure S6.** (a) Emission spectra and (b) fluorescent color change of **QP** 10 µ*M* in different fractions of water in a THF-water mixture.

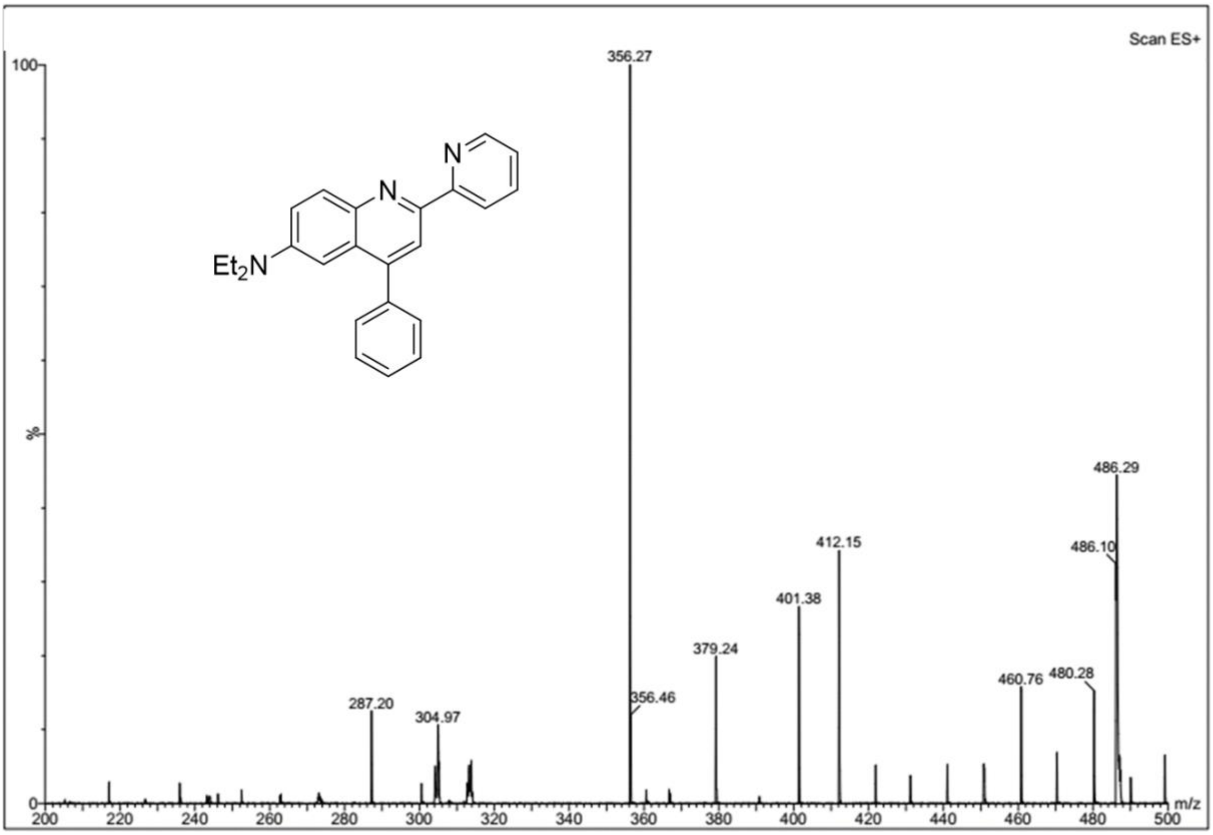


**Figure S1.** ESI-MS spectra of **QP**.

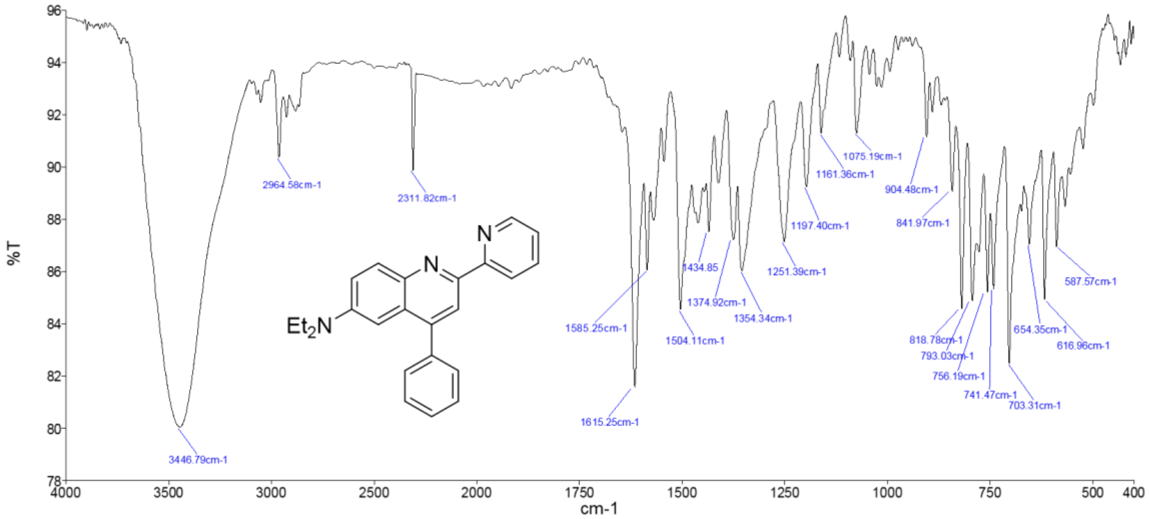


**Figure S2.** FT-IR spectra of **QP**.

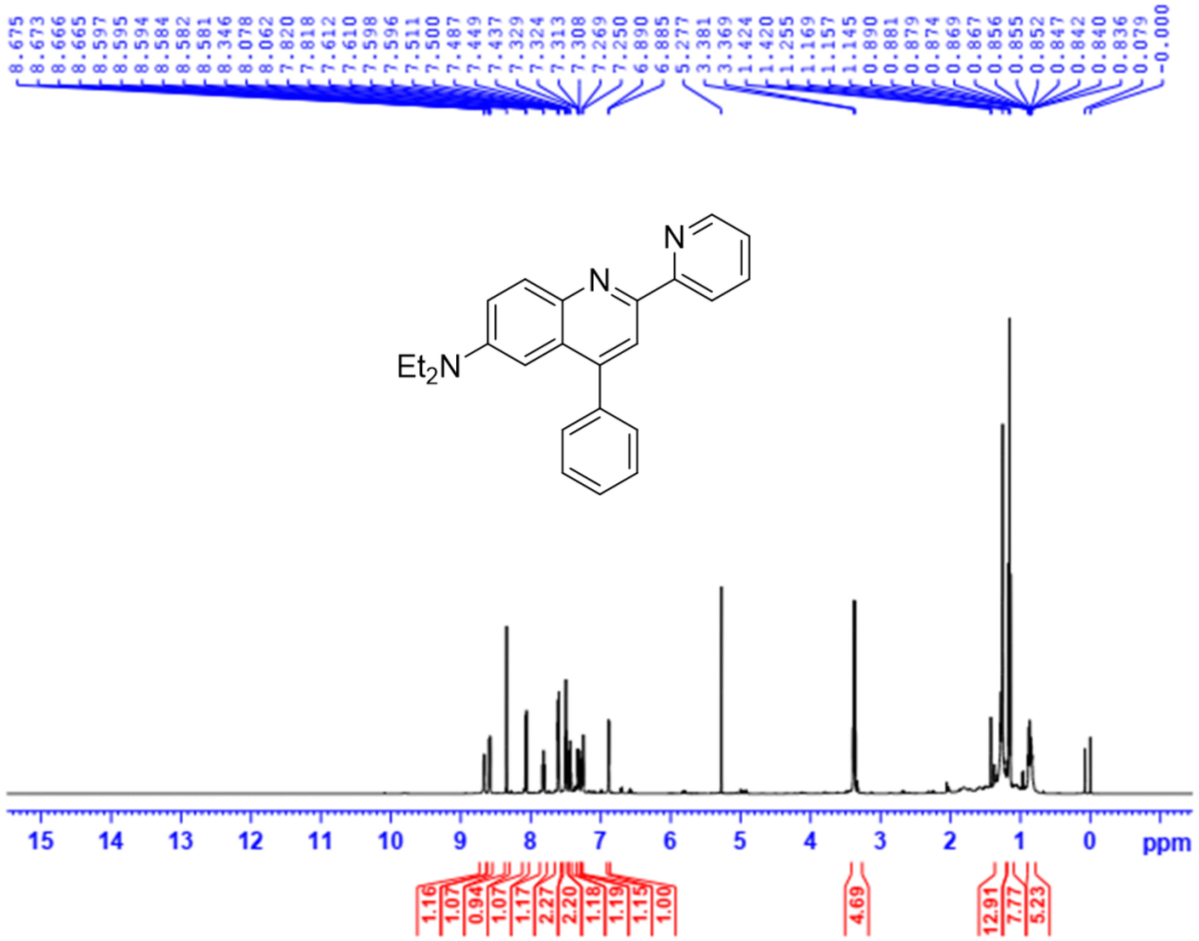


**Figure S3.** $^{1}$H NMR spectra of **QP** in $CDCl_3$.

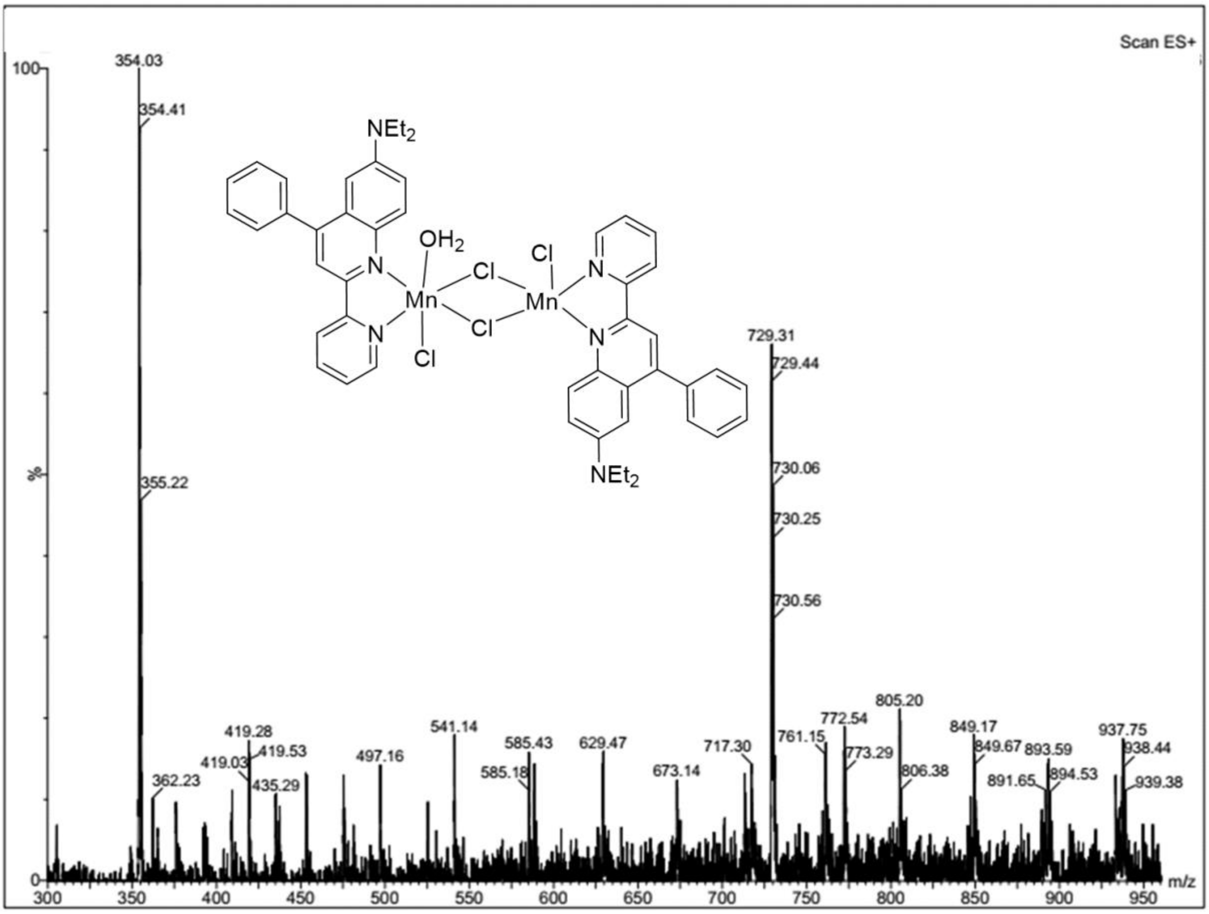


**Figure S4.** ESI-MS spectra of **MnQP**.

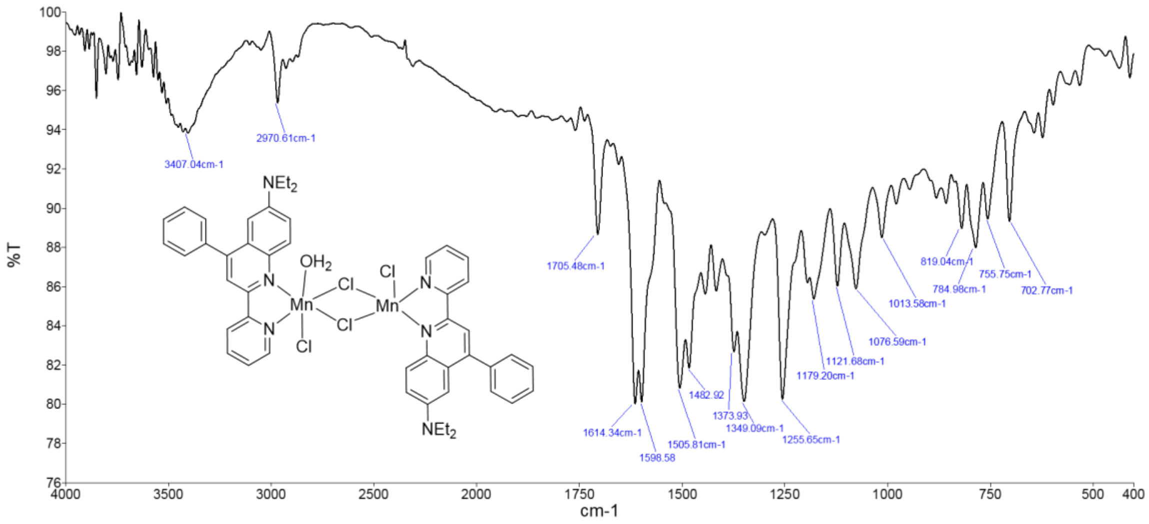


**Figure S5.** FT-IR spectra of **MnQP**.

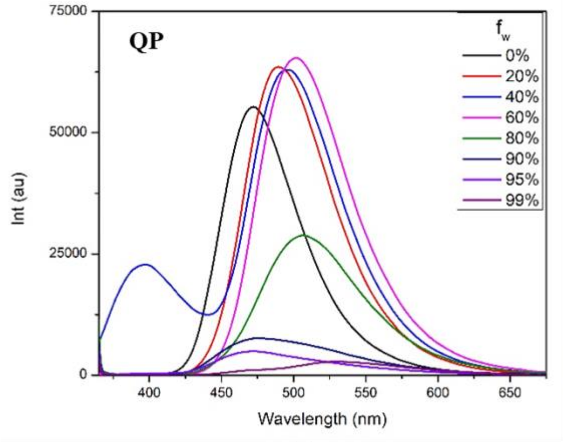


(a)

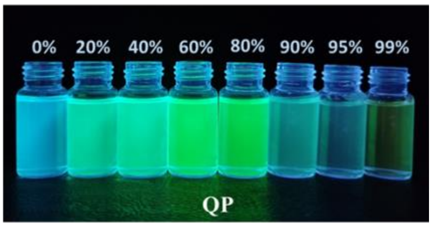


(b)

**Figure S6.** (a) Emission spectra and (b) fluorescent color change of **QP** 10 μ*M* in different fraction of water in a THF-water mixture.
